# Are all negative words alike? Behavioral differences in processing negative words associated and not associated with physical and social pain

**DOI:** 10.3389/fpsyg.2024.1474945

**Published:** 2024-09-30

**Authors:** Eleonora Borelli, Francesca Pesciarelli

**Affiliations:** ^1^Department of Medical and Surgical Sciences, University of Modena and Reggio Emilia, Modena, Italy; ^2^Department of Biomedical, Metabolic and Neural Sciences, University of Modena and Reggio Emilia, Modena, Italy

**Keywords:** word processing, affect, semantics, physical pain, social pain, reaction time, motor response

## Abstract

Studies examining whether stimulus valence affects cognitive processing and motor responses yield mixed results, possibly due to treating negative words as a homogeneous category. Words related to pain may hold distinct status because of their relevance to survival. Thus, they offer a unique opportunity to investigate semantic influences on cognitive processing. This study aims to determine if words related to physical and social pain elicit stronger aversion than general negative words by assessing the Affective Compatibility Effect in implicit and explicit tasks. In Experiment 1, 35 participants performed a lexical decision task on 60 positive words and 60 negative words, of which 20 not related to pain, 20 related to physical pain, and 20 related to social pain. Participants held down the central key of a keyboard and released it to press a key far from the screen (avoidance condition) or close to the screen (approach condition) for words. In Experiment 2, 43 participants performed a valence evaluation task on the same words. They held down the central key and released it to press a key close to the screen for positive words and a key far from the screen for negative words (congruent condition), or the opposite (incongruent condition). In Experiment 1, we found faster RTs for social pain-related words compared to other categories. We also found faster RTs in the approach condition than in the avoidance condition, regardless of whether valence or semantics were considered as independent variables. In Experiment 2, we found faster RTs in the congruent condition than in the incongruent condition when semantics was considered as independent variable. We also found an interaction valence*condition, with faster RTs for negative words in the congruent condition than in the incongruent condition when valence was considered as independent variable. Our findings suggest that, notwithstanding pain-related words do not affect aversive behaviors compared to negative, pain-unrelated words, they are processed faster when conveying social pain. This supports the hypothesis that the cognitive system differentiates and responds congruently not only based on general semantic categories, like pain, but also possibly based on nuances within it.

## 1 Introduction

In contemporary studies of human emotion, it is widely accepted that stimuli are automatically evaluated in terms of their affective valence, i.e., their degree of pleasantness or unpleasantness, along a negative-to-positive gradient (Citron et al., 2016). The assessment of stimuli in such terms is highly relevant for survival, as humans must rapidly detect, process, and react to stimuli associated with potentially rewarding or threatening events. Efforts have been made by scholars to investigate whether the valence degree associated with a stimulus affects its processing, specifically determining if one valence leads to a processing advantage over the other.

Concerning word processing, data show some inconsistencies, suggesting that positive words are processed more effectively than or comparably to negative words, with less evidence supporting the opposite, once various experimental factors and stimulus properties are taken into account (Kauschke et al., 2019; Hinojosa et al., 2020; Barriga-Paulino et al., 2022). One of the reasons for these inconsistencies may stem from the fact that studies on valenced stimuli have predominantly treated positive and negative words as unitary categories. Only recently, it has been suggested that positive and negative stimuli may not be singular classes, as indirectly demonstrated by various studies on patients with phobias, anxiety, or depression using categories of stimuli relevant to the individuals (Abado et al., 2020). Rather, the behavioral and neural correlates of processing valenced stimuli may differ based on their specific semantic content (Wurm, 2007; Witherell et al., 2012; Kveraga et al., 2015; Lindquist et al., 2016; Gilioli et al., 2023a,b). For this reason, it is possible to speculate that when the cognitive system needs to determine the priority of a stimulus, its valence interacts with its semantic content, generating a specific response.

Pain words belong to the realm of negative words. Given the high relevance of pain experiences to everyday life and survival, one might wonder whether the words we use for conveying pain may have a specific status and may be perceived as more negative than merely negative words. The results of a normed database of words associated with physical and social pain suggest that this indeed may be the case (Borelli et al., 2018). The strength of association of negative words to pain affected their perceived valence in that not all words were rated as similarly negative. Rather the more a word was associated with pain, the more negative it was rated. Notably, social pain words, i.e., words that evoke painful feelings associated with actual or potential social rejection, exclusion, or loss (Eisenberger and Lieberman, 2004; MacDonald and Leary, 2005), were rated as more negative, pain-related, and reflecting more intense and unpleasant pain experiences than physical pain words (Borelli et al., 2018, 2021). Although pain is not commonly classified as an emotion in itself, it is regarded as an emotional experience in addition to being a sensory one, characterized by its unpleasant nature (Raja et al., 2020). Hence, pain words could serve as a suitable model for investigating whether the specificity of a stimulus’s semantic content in the environment can affect its processing beyond its negative valence (Gilioli et al., 2023a,b).

Valence processing triggers tendencies for appetitive and aversive behaviors. Specifically, processing a negative stimulus activates a withdrawal-aversive system, responsible for escaping from a potentially harmful context or keeping a stimulus away from oneself, while processing a positive stimulus activates an approach-appetitive system, responsible for seizing an opportunity or acting toward a positive event. Adaptive approach and avoidance to both reward and threat are essential for averting harm and promoting well-being. To study if the congruency between the valence stimulus and the appropriate response results in a facilitation on behavioral measures (Affective Compatibility Effect; Solarz, 1960), researchers use paradigms in which participants respond as fast as possible with simple approach and avoidance movements to affective stimuli.

The purpose of the present study was to examine whether, given the same valence, the semantics of words affects their cognitive processing differently. To this aim, we explored if words related to physical and social pain elicit greater aversion compared to merely negative words. This was measured by assessing the Affective Compatibility Effect in an approach/avoidance paradigm with an implicit task (lexical decision task) and an explicit task (valence evaluative task).

## 2 Experiment 1

### 2.1 Materials and methods

#### 2.1.1 Participants

A total of 35 healthy participants (21 females and 14 males; age range: 19–28 years old; mean age: 20.8; SD: 2.4) took part in the study after informed consent. Inclusion criteria were being Italian native speakers, right-handed, with normal or corrected-to-normal vision, with no history of neurological or psychiatric illnesses, and not under medication affecting mood or pain perception at the time of the study. Handedness was assessed using the Edinburgh Inventory (Oldfield, 1971). Participants who were students at the University of Modena and Reggio Emilia received academic credits for their participation. The sample size was determined using a heuristic approach, based on typical participant numbers commonly employed in studies within this field (Lakens, 2022).

The study was conducted according to the recommendations of the Italian Association of Psychology ethical guidelines and with the standard ethical procedures of the University of Modena and Reggio Emilia. All subjects gave written informed consent in accordance with the Declaration of Helsinki.

#### 2.1.2 Stimuli

Stimuli comprised 120 Italian words and 120 pseudo-words generated using Wuggy (Keuleers and Brysbaert, 2010). Italian words belonged to the following three categories: 60 positive words, referred to as PosW (e.g., *rispetto*, respect) and 60 negative words, referred to as NegW, of which 20 pain-unrelated (NegNoPW; e.g., *ignoranza*, ignorance), 20 related to physical pain (PhysPW, e.g., *emicrania*, cephalgy), and 20 related to social pain (SocPW, e.g., *tradimento*, betrayal). PosW and NegNoPW were selected from the Italian version of the ANEW database (Affective Norms for English Words; Montefinese et al., 2014), while PhysPW and SocPW were selected from the Italian WOP database (Words of Pain; Borelli et al., 2018). As the ANEW and WOP databases are well-established, widely used and validated resources for psycholinguistic, affective and pain-related word norms, a preliminary validation study of the stimuli was not considered necessary. Words in the different conditions were chosen so that they were balanced for the main psycholinguistic, distributional, affective, and pain-related variables that are known to affect linguistic comprehension processes. Specifically, PosW and NegW, which differed for valence [*F*(1,118) = 2,752, *p* < 0.001], were balanced for length in letters, frequency, familiarity, age of acquisition, imageability, concreteness, context availability, and arousal. NegNoPW, PhysPW, and SocPW were balanced for length in letters, frequency, familiarity, age of acquisition, context availability, valence, and arousal, but not for imageability [*F*(2,57) = 4.805, *p* = 0.012], with SocPW significantly less imaginable than PhysPW, and concreteness [*H*(2) = 23.459, *p* < 0.001], with PhysPW significantly more concrete than NegNoPW and SocPW. PhysPW and SocPW were also balanced for pain relatedness, pain intensity, and pain unpleasantness. The list of words is available as Supplementary material.

All pseudo-words were created with legal combinations of letters and differed by one to three letters from each of the 120 Italian words.

#### 2.1.3 Procedure

**Figure 1:**
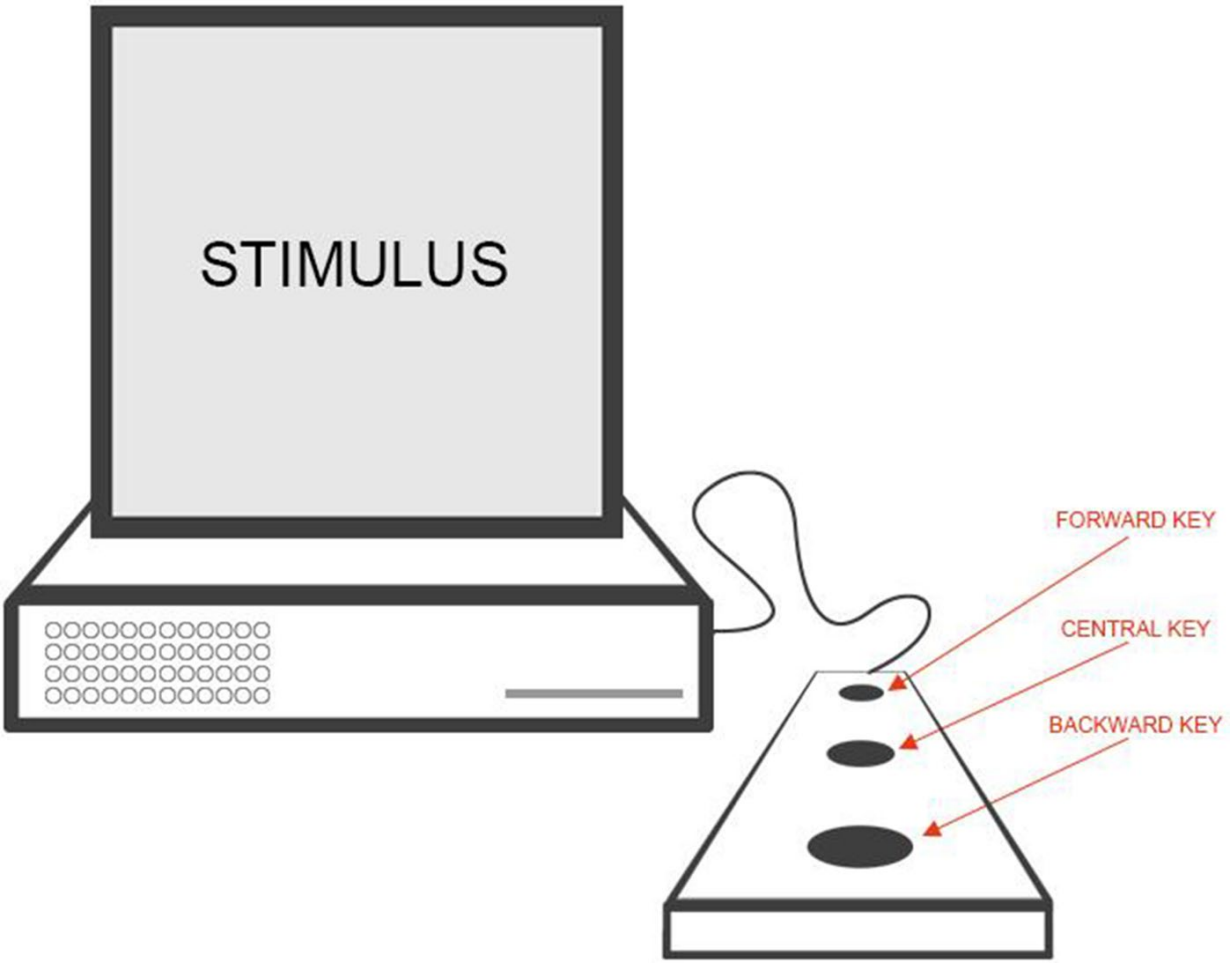
Schematic representation of the three-button response box. The response box was positioned lengthwise on the right side of the participant. The three keys were evenly spaced: one located at the end near the participant (backward key), another at the opposite end (forward key) near the monitor, and a centrally located one between them (central key). The keys were enlarged and designed to be pressed using the palm of the hand.

E-Prime 2.0 (Psychology Software Tools, Pittsburgh, PA, United States) was used for stimulus presentation and behavioral response collection. Participants were comfortably seated in front of a monitor, positioned approximately 70 cm away from their eyes, within a dimly illuminated and soundproof room. A three-button response box was positioned lengthwise on their right side. The three keys were evenly spaced: one positioned at the end of the keyboard near the participant, another positioned at the opposite end near the monitor, and a centrally located one between them. Additionally, the keys were enlarged and were to be pressed using the palm of the hand (Figure 1).

Task instructions were provided by the experimenter at the beginning of the experiment and displayed on the monitor prior to each trial as a reminder. Participants were instructed to look at a fixation cross (+) in the center of the screen while holding down the central key of the response box. The fixation cross, which lasted 450 ms, was then replaced by a letter string. The appearance of the letter string (white, non-capitalized letters, 24-point Arial bold font, against a black background) varied randomly between 1,000 and 2,000 ms to prevent anticipation of the response. At the appearance of the letter string, participants had to release the central key and press down, as quickly and accurately as possible, one of the other two keys or refrain from responding based on whether the letter string constituted a word or a pseudo-word, respectively (implicit, lexical decision, and go/nogo task). Specifically, in the avoidance condition (i.e., flexion of the arm), they were instructed to press the key far from the screen and in the approach condition (i.e., extension of the arm) they were instructed to press the key close to the screen. A response time limit of 2,500 ms was imposed. Following each trial, the screen remained blank for 1,000 ms before the commencement of the subsequent trial. Response accuracy and key release reaction rimes (RTs) were recorded for each trial. Instructions were explicit-converted, i.e., set up to induce a stimulus-centered perspective in approach/avoidance responses (Phaf et al., 2014).

The experimental stimuli were presented twice, once for each condition. The avoidance and approach conditions constituted two separate, consecutive blocks. The order of word presentation was pseudo-randomized within each block, and the sequence of the two blocks was counterbalanced across participants. A practice session with words and pseudo-words not included in the stimulus list preceded each block.

The participants were informed that after the experiment they would perform a free recall task to ensure they maintained attention throughout the entire task. The overall duration of the experiment was 1 h.

#### 2.1.4 Analysis

Inaccurate responses, defined as those trials where a word was erroneously evaluated as a pseudo-word (no central key release) or a pseudo-word was erroneously evaluated as a word (incorrect response key press), were removed from the dataset.

Pseudo-words were removed from the dataset. Key release reaction times (RTs) below 300 ms or above 1,500 ms were removed from the analysis. The cutoffs were determined based on three key criteria: first, the observation that key release tends to be slightly slower than key press (Bjørklund, 1991); second, the typical cutoffs used in studies involving lexical decision tasks and valence evaluations; and third, a visual inspection of the distribution of our data.

The non-normality of the key release RT distribution was assessed using a Shapiro test. Due to this non-normality, a non-parametric Aligned Rank Transform (ART) ANOVA was conducted on key release RTs with Valence (PosW vs. NegW) and Condition (avoidance vs. approach) as within-subject factors. A second ART ANOVA was conducted with Semantics (PosW vs. NegNoPW vs. PhysPW vs. SocPW) and Condition (avoidance vs. approach) as within-subject factors. Tukey-corrected *post-hoc* tests were conducted on significant main effects and interactions.

Statistical analyses were performed with R version 4.3.3 (R Core Team, 2024). In accordance with standard scientific practice, the significance level (*α*) was set at 0.05 and only statistically significant results were reported.

### 2.2 Results

A total of 1.4% of trials were removed from the dataset due to inaccuracy. The final sample accuracy ranged from 95.6 to 99.8%. Given the low rates of inaccurate data, likely due to a ceiling effect, further analysis on inaccuracy was not pursued.

A total of 0.7% of trials were removed from the dataset because they fell outside the 300–1,500 ms cut-off.

**Figure 2:**
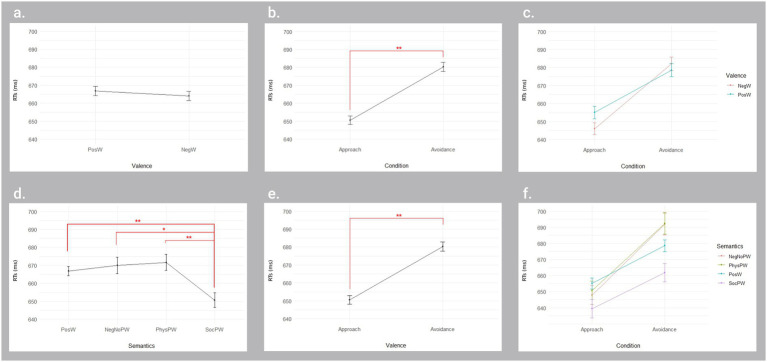
Significant and non-significant main and interaction effects from the ART ANOVAs on key release reaction times for Valence × Condition and Semantics × Condition in the lexical decision task. The upper panel displays the results for the Valence × Condition ANOVA, indicating no significant main effect of Valence **(A)**, but a significant main effect of Condition **(B)**. The interaction effect was also not significant **(C)**. The lower panel shows the results for the Semantics × Condition ANOVA, indicating a significant main effect of Semantics **(D)** and Condition **(E)**, but not for the interaction **(F)**. Error bars represent standard error. RTs, Reaction times; ms, milliseconds; PosW, Positive words; NegW, Negative words; NegNoPW, Negative pain-unrelated words; PhysPW, Physical pain-related words; SocPW, Social pain-related words. *p < 0.05; **p < 0.01.

The ART ANOVA on accurate key release RTs on Valence and Condition revealed a main effect for Condition [*F*(1, 8,296) = 76.761, *p* < 0.001], with significantly faster RTs in the approach condition (651 ms) than in the avoidance condition (680 ms) (Figure 2).

The ART ANOVA on Semantics and Condition revealed a main effect for Semantics [*F*(3, 8,292) = 5.07, *p* = 0.002], with significantly faster RTs for SocPW (651 ms) than PosW (667 ms; *p* = 0.001), NegNoPW (670 ms; *p* = 0.02), and PhysPW (672 ms, *p* = 0.002), and a main effect for Condition [*F*(1, 8,292) = 59.5016, *p* < 0.001], with significantly faster RTs in the approach condition (651 ms) than in the avoidance condition (680 ms) (Figure 2).

## 3 Experiment 2

### 3.1 Materials and methods

#### 3.1.1 Participants

A total of 43 healthy participants (28 females and 15 males; age range: 19–26 years old; mean age: 20.5; SD: 1.9) took part in the study after informed consent. The sample size determination approach, inclusion criteria, handedness assessment, and reward were the same as in Experiment 1.

The study was conducted according to the recommendations of the Italian Association of Psychology ethical guidelines and with the standard ethical procedures of the University of Modena and Reggio Emilia. All subjects gave written informed consent in accordance with the Declaration of Helsinki.

#### 3.1.2 Stimuli

Stimuli comprised 120 Italian words from Experiment 1 (60 PosW and 60 NegW of which 20 NegNoPW, 20 PhysPW, and 20 SocPW).

#### 3.1.3 Procedure

E-Prime 2.0 (Psychology Software Tools, Pittsburgh, PA, United States) was used for stimulus presentation and behavioral response collection. The experimental procedure remained consistent with that of Experiment 1. However, in Experiment 2, at the appearance of the word, participants had to release the central key and press down, as quickly and accurately as possible, one of the other two keys based on word’s valence (explicit, valence evaluation task). Specifically, in the congruent condition, they were instructed to press the key close to the screen for positive words (approach, i.e., extension of the arm) and the key far from the screen for negative words (avoidance, i.e., flexion of the arm). In the incongruent condition, they were instructed to press the key far from the screen for positive words (avoidance, i.e., flexion of the arm) and the key close to the screen for negative words (approach, i.e., extension of the arm).

Differently from Experiment 1, no time limit for the response was imposed.

The overall duration of the experiment was 1 h.

#### 3.1.4 Analysis

Inaccurate responses, defined as those trials where word valence was not evaluated (no central key release) or was evaluated incorrectly (incorrect response key press), were removed from the dataset.

Key release RTs below 300 ms or above 1,500 ms were removed from the analysis, as per Experiment 1.

The non-normality of the key release RT distribution was assessed using a Shapiro test. Due to this non-normality, a non-parametric ART ANOVA was conducted on key release RTs with Valence (PosW vs. NegW) and Condition (congruent vs. incongruent) as within-subject factors. A second ART ANOVA was conducted on key release RTs with Semantics (PosW vs. NegNoPW vs. PhysPW vs. SocPW) and Condition (congruent vs. incongruent) as within-subject factors. Tukey-corrected post-hoc tests were conducted on significant main effects and interactions.

Statistical analyses were performed with R version 4.3.3 (R Core Team, 2024). In accordance with standard scientific practice, the significance level (*α*) was set at 0.05 and only statistically significant results were reported.

### 3.2 Results

A total of 4.9% of trials were removed from the dataset due to inaccuracy. A participant was identified with notably low accuracy (0.8%). Upon reviewing the original dataset, it appeared likely that the participant had inadvertently responded by reversing the keys. As a result, she was excluded from the analyses. The final sample was composed of 42 participants (27 females and 15 males; age range: 19–26 years; mean age: 20.6; SD: 1.9), whose accuracy ranged from 87.9 to 100%. Given the low rates of inaccurate data, likely due to a ceiling effect, further analysis on inaccuracy was not pursued. A total of 1.3% of trials were removed from the dataset because they fell outside the 300–1,500 ms cut-off.

**Figure 3:**
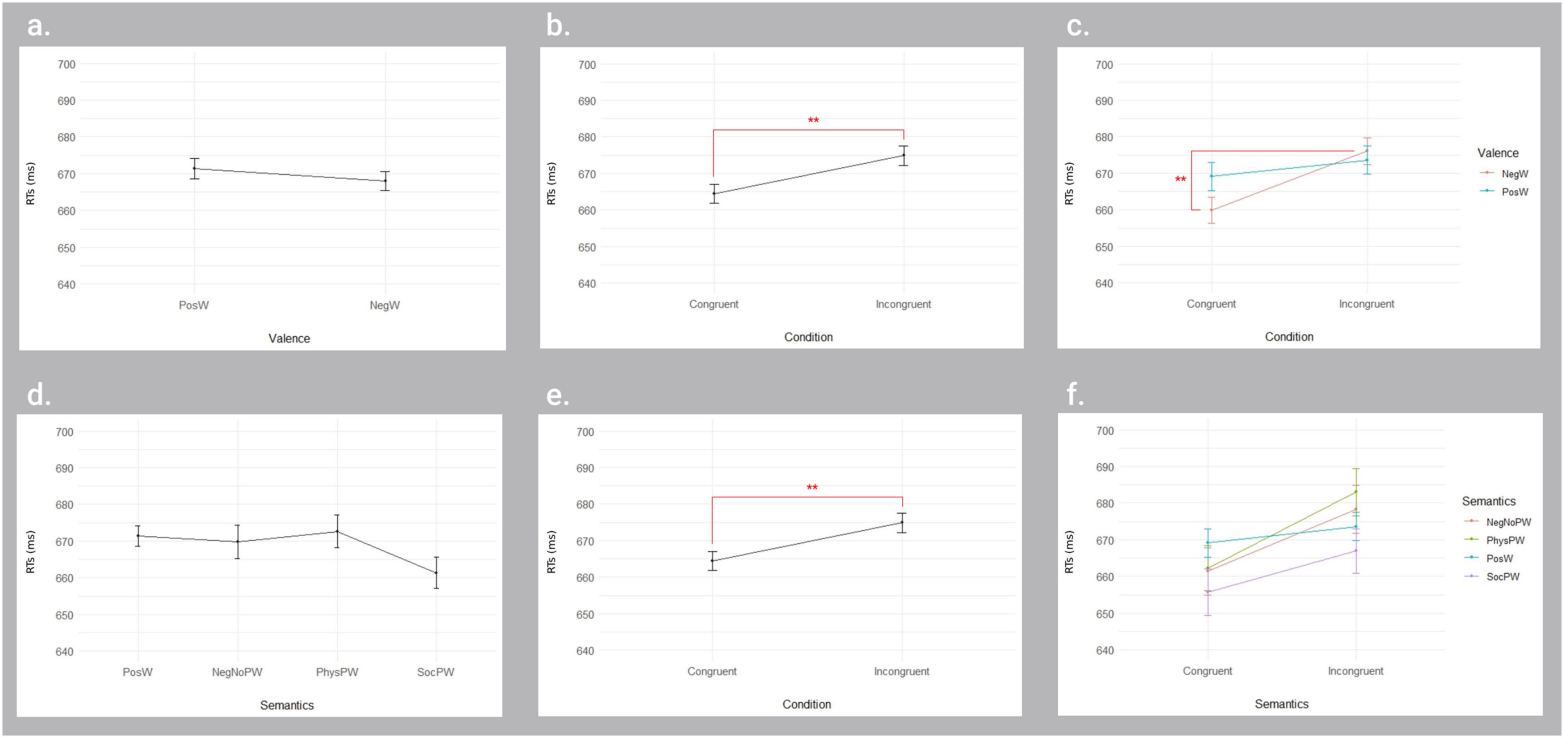
Significant and non-significant main and interaction effects from the ART ANOVAs on key release reaction times for Valence × Condition and Semantics × Condition in the lexical decision task. The upper panel displays the results for the Valence × Condition ANOVA, indicating no significant main effect of Valence **(A)**, but a significant main effect of Condition **(B)** and a significant interaction Valence × Condition **(C)**. The lower panel shows the results for the Semantics × Condition ANOVA, indicating no significant main effect of Semantics **(D)**, but a significant main effect of Condition **(E)**. Error bars represent standard error. The interaction effect was also not significant **(F)**. RTs, Reaction times; ms, milliseconds; PosW, Positive words; NegW, Negative words; NegNoPW, Negative pain-unrelated words; PhysPW, Physical pain-related words; SocPW, Social pain-related words. *p < 0.05; **p < 0.01.

The ART ANOVA on Valence and Condition revealed a main effect for Condition [*F*(1, 9,678) = 7.847, *p* = 0.005], with significantly faster RTs in the congruent condition (664 ms) than in the incongruent condition (675 ms), and a significant interaction Valence*Condition [*F*(1, 9,678) = 4.454, *p* = 0.035], with significantly faster RTs for NegW in the congruent condition (660 ms) than in the incongruent condition (676 ms, *p* < 0.001) (Figure 3).

The ART ANOVA on Semantics and Condition revealed a main effect for Condition [*F*(1, 9,674) = 7.128, *p* = 0.008], with significantly faster RTs in the congruent condition (664 ms) than in the incongruent condition (675 ms) (Figure 3).

## 4 Discussion

The main result we found in this study was an advantage in processing SocPW compared to PosW, NegNoPW, and PhysPW, regardless of the motor tendency, confirming that negative words are treated differently by the cognitive system based on their semantics.

Literature is recently suggesting that semantic content should be included into models of word processing together with valence. A recent meta-analysis of 397 fMRI studies (Lindquist et al., 2016) found that there is no consistent voxel pattern that universally represents negative stimuli. Although people find a range of stimuli unpleasant, the brain does not process all negative stimuli in the same way. This is evident in everyday behavior: while few are inclined to approach a dangerous animal or an armed person, the common occurrence of gawking at accident scenes suggests that people are often irresistibly compelled to observe certain negative events (Kveraga et al., 2015; Brooks et al., 2016). This depends on several factors, with context being one of the most important (Holt et al., 2009; Hauser and Schwarz, 2023).

The key role of semantics, even when valence is held constant, has also been confirmed using pain-related words as a model. Although driven by different research inquiries, Thomas Weiss and his research group have made significant contributions in this field. In their initial studies, they discovered that pain-related semantic primes might activate neural networks involved in pain memory and processing in both healthy participants and migraine patients compared to neutral primes (Dillmann et al., 2000; Weiss et al., 2003). The authors later began using negative, pain-unrelated words as a control condition to ensure that the observed effects were attributable to the semantic content associated with pain rather than the negative valence. They still found that processing pain-related words, as opposed to pain-unrelated words, could not be solely explained by their emotional valence level (Richter et al., 2010; Eck et al., 2011; Ritter et al., 2016, 2019). Furthermore, despite pain perception being enhanced regardless of whether the primes were negative and associated with pain or not (Richter et al., 2014), the rating of valenced words (but not neutral ones) was decreased by painful stimuli (Brodhun et al., 2021). This effect was more pronounced for negative, pain-related than for negative, pain-unrelated words. Their findings also showed that processing pain-related words involves areas thought to be involved in experiencing physical pain, even in the absence of painful stimulation, or it primes their activation in the presence of painful stimulation (Richter et al., 2010; Eck et al., 2011; Ritter et al., 2016, 2019), as also found by others (Knost et al., 1997; Osaka et al., 2004; Nikendei et al., 2005; Gu and Han, 2007; Kelly et al., 2007; Sitges et al., 2007; Gilioli et al., 2023a).

Thus, the differences in processing we observed between negative pain-related words and negative pain-unrelated words are consistent with previous research. Although this finding might be expected, what is particularly intriguing in our study is the ability of the cognitive system to discriminate nuances within the same semantic category. Notably, this pattern persisted across both tasks, although it only reached statistical significance in the lexical decision task (Experiment 1). To the best of our knowledge, there are currently no studies that explicitly compare behavioral responses between words associated with physical pain and those associated with social pain. However, there is some evidence in the literature to suggest that they are two distinct concepts, both cognitively and emotionally, and that social pain tends to be perceived as more relevant than physical pain. For example, nearly 75% of people identified the loss of a close relationship, whether due to death or breakup, as the most negative emotional event they had ever experienced (Holmes and Rahe, 1967; Jaremka et al., 2011). While emotional pain can persist for long periods of time, the sensory experiences associated with physical pain typically do not last beyond the acute phase of the painful event (Meyer et al., 2015). This distinction appears to be maintained when the two types of pain are translated into words. The psycholinguistic and emotional norms of words associated with physical and social pain in different populations revealed a consistent pattern in which words associated with social pain were perceived as more negative and semantically closer to the concept of pain than those associated with physical pain (Borelli et al., 2018, 2021). Furthermore, words associated with social pain appeared to engage different neural mechanisms compared to words associated with physical pain (Borelli et al., 2023). Specifically, compared to neural activations associated with nociceptive pain stimulation, words associated with social pain elicited widespread activation across regions within the pain matrix, consistent with those involved in the affective-motivational aspects of nociception. Conversely, words associated with physical pain elicited activation in a limited cluster of regions primarily associated with sensory-discriminative aspects.

We did not observe any significant differences between positive and negative words. It could be hypothesized that the unique effect of the semantic content of SocPW on word processing may not be statistically apparent when this specific category is analyzed in aggregate with other negative words with mixed semantic meanings, as was the case with our 20 negative pain-unrelated words, potentially leading to the erroneous conclusions.

Notably, the cognitive processing facilitation for SocPW was only observed in the lexical decision task. The lexical decision task requires access to the semantics of the word, but studies have shown that it can be performed correctly without access its affective connotation (Hinojosa et al., 2010; Citron et al., 2016). Conversely, access to the affective connotation prior to word semantics of the word is implausible. This suggests that the valence evaluation task requires an initial access to the semantics in order to evaluate the valence. Comparing the RTs of the lexical decision task and the valence evaluation task for each semantic category, we see that they are quite similar for PosW (667 vs. 672 ms), NegNoPW (670 vs. 670 ms), and PhysPW (672 vs. 673 ms). However, they are slightly slower for SocPW in the valence evaluation task (661 ms) compared to the lexical decision task (651 ms), although the difference does not reach significance in a *t*-test (*p* = 0.07). Thus, assigning a valence to words whose semantics prioritizes their processing, such as SocPW, seems to slow down the RTs. According to the Automatic Vigilance Hypothesis, negative stimuli are attended preferentially because failure to avoid a negative stimulus can have dire consequences (Pratto and John, 1991; Taylor, 1991; Wentura et al., 2000). Greater attentional commitment to negative stimulus features may divert cognitive resources away from processing other non-affective items’ properties, resulting in slower RTs in many non-affective tasks (Gao et al., 2022). However, if we recognize the same role for the semantics, it can be hypothesized that the identification of negative valence seizing resources for the execution of tasks on other properties could also apply to semantics itself at the expense of valence. Since access to semantic meaning occurs before access to valence, the detection of semantic meaning would facilitate the response in a lexical decision task, whereas the requirement to evaluate its valence could be slowed down by the allocation of cognitive resources to the semantic aspects of the stimulus or by a delayed disengagement from the semantic content. In other words, we respond quickly to an alarming semantics, which then slows us down in making a valence judgment. However, this result can also be interpreted from a different perspective. In our experiment, the SocPW were less imaginable and less concrete than the other categories, a characteristic that should theoretically disadvantage them in terms of semantic accessibility and RTs. Nevertheless, social pain words, like other abstract words, are primarily grounded in internal affective states and are semantically more interconnected than concrete words (such as our PhysPW), which are more closely tied to sensory-motor experiences (Kousta et al., 2011; Vigliocco et al., 2013). This could have been resulted in a processing advantage for SocPW over PhysPW in a semantic task.

Another interesting result from our study is that the Affective Compatibility Effect was reversed in the lexical decision task, with faster RTs when approaching negative stimuli (and avoiding positive ones) and slower RTs when avoiding negative stimuli (and approaching positive ones), but not in the valence evaluation task, with faster RTs when avoiding negative stimuli and slower RTs when approaching them. One possible explanation, that has been recently tested (Ballotta et al., under review), involves the influence of emotional stimuli on attentional allocation, suggesting a compatibility effect between the direction of attentional shift induced by valence stimuli. In particular, negative stimuli may affect attentional allocation in a paradoxical way: an initial phase of attentional capture by the negative stimulus may be rapidly followed by a withdrawal of attention away from the stimulus. This process is consistent with the behavioral pattern of an approach response followed by an avoidance response. Translating this attentional pattern into motor terms, the initial capture of the semantic meaning of SocPW may elicit an approach movement. Conversely, the subsequent withdrawal of attention, corresponding to the identification of its valence, may trigger an avoidance movement. However, the Affective Compatibility Effect on approach/avoidance to valenced stimuli was not the aim of the present study, which was not designed to optimally test this process. We believe that the moderating variables that interfere with the Affective Compatibility Effect on approach/avoidance tasks are not yet sufficiently understood to design a study that can draw definitive conclusions about the dynamics of this process (Phaf et al., 2014). We tried to control for these external variables as much as possible. For example, we formatted the instructions to be implicit (lexical decision task) and explicit (valence evaluation task), and we used a within-subjects design to also test the opposite condition (Phaf et al., 2014). However, we prefer to refrain from further commenting on this result, except to note that no interaction between the semantics of the words and the congruence of approach/avoidance movements was observed.

A strength of our study, in contrast to others in the field, is the careful selection of stimuli. While many studies typically balance words on the basis of frequency, length, valence, and arousal, our approach involved balancing a wider range of distributional, psycholinguistic, and emotional variables, in addition to those associated with pain as identified in the WOP database (Borelli et al., 2018). It is worth noting, however, that achieving a balance between imageability (Paivio et al., 1968; Paivio, 2013b) and concreteness (Paivio and Begg, 1971; Paivio, 2013a) proved unattainable in our study. That these two variables were not balanced was expected. Words associated with social pain tend to be less concrete compared to those associated with physical pain, and it is reasonable to speculate that they are also less concrete compared to a generic category of negative words not associated with pain, which likely contains both more and less concrete terms (Borelli et al., 2018). These observations may be extended to imageability as well, given the strong relationship between the two variables (Paivio et al., 1968; Gilhooly and Logie, 1980; Soares et al., 2012; Guasch et al., 2016; Yee, 2017). Because it was not possible to include them as covariates, as they were not anticipated in the type of analysis conducted, further testing is needed to investigate whether our results can be explained by their effects.

A similar issue must also be acknowledged with regard to gender. Since covariates could not be tested in the type of analysis we conducted, the imbalance in the proportion of males and females in our experiments presents a potential limitation. We run a Wilcoxon rank-sum test to compare RTs between males and females that showed that females were significantly faster than males (654 ms vs. 683 ms, respectively; *p* < 0.001). Given that gender appears to play a modulatory role in our and others’ studies on pain perception (Borelli et al., 2023), future research should take this variable into account.

In conclusion, our results align with studies that underscore the importance of moving beyond treating negative words as a monolithic category and instead considering the specific semantic nuances that shape their processing in the brain. By transcending a one-size-fits-all approach to negative words, researchers can uncover the intricate cognitive mechanisms underlying the processing of words that share the same valence but differ in semantic content.

## Data Availability

The raw data supporting the conclusions of this article will be made available by the authors, without undue reservation.
