# Supplementary material for: Are all negative words alike? Behavioral differences in processing negative words associated and not associated with physical and social pain
Source: Front Psychol. 2024 Sep 30;15:1474945. doi: 10.3389/fpsyg.2024.1474945 (PMC11472825; doi:10.3389/fpsyg.2024.1474945)
Supplement: Supplementary file 1 [file Table_1.docx]

**Supplementary Table 1.** List of Italian words utilized in the study alongside their English translations (translated via ChatGPT) and the semantic categories to which they belong.

| Semantics | Italian words | English translation |
| --- | --- | --- |
| NegNoPainW | Alcolista | Alcoholic |
| NegNoPainW | Astio | Animosity |
| NegNoPainW | Avversità | Adversity |
| NegNoPainW | Collera | Anger |
| NegNoPainW | Debito | Debt |
| NegNoPainW | Discarica | Dump |
| NegNoPainW | Feccia | Scum |
| NegNoPainW | Ignoranza | Ignorance |
| NegNoPainW | Immondizia | Garbage |
| NegNoPainW | Incubo | Nightmare |
| NegNoPainW | Minaccia | Threat |
| NegNoPainW | Miseria | Misery |
| NegNoPainW | Negligenza | Negligence |
| NegNoPainW | Obesità | Obesity |
| NegNoPainW | Penalità | Penalty |
| NegNoPainW | Sepoltura | Burial |
| NegNoPainW | Terrorista | Terrorist |
| NegNoPainW | Tragedia | Tragedy |
| NegNoPainW | Truffa | Scam |
| NegNoPainW | Vandalo | Vandal |
| PhysPW | Artrite | Arthritis |
| PhysPW | Emicrania | Migraine |
| PhysPW | Ferita | Wound |
| PhysPW | Frattura | Fracture |
| PhysPW | Frustata | Whipping |
| PhysPW | Infarto | Heart attack |
| PhysPW | Infermità | Ailment |
| PhysPW | Infezione | Infection |
| PhysPW | Lacerazione | Laceration |
| PhysPW | Lesion | Injury |
| PhysPW | Mutilazione | Mutilation |
| PhysPW | Nevralgia | Neuralgia |
| PhysPW | Paralisi | Paralysis |
| PhysPW | Piaga | Sore |
| PhysPW | Proiettile | Bullet |
| PhysPW | Soffocamento | Suffocation |
| PhysPW | Tortura | Torture |
| PhysPW | Ulcera | Ulcer |
| PhysPW | Ustione | Burn |
| PhysPW | Vomito | Vomit |
| SocPW | Abbandono | Abandonment |
| SocPW | Aborto | Abortion |
| SocPW | Abuso | Abuse |
| SocPW | Addio | Farewell |
| SocPW | Crepacuore | Heartbreak |
| SocPW | Depression | Depression |
| SocPW | Disperazione | Desperation |
| SocPW | Fallimento | Failure |
| SocPW | Infelicità | Unhappiness |
| SocPW | Lacrima | Tear |
| SocPW | Lutto | Mourning |
| SocPW | Patimento | Suffering |
| SocPW | Perdita | Loss |
| SocPW | Separazione | Separation |
| SocPW | Strazio | Anguish |
| SocPW | Stress | Stress |
| SocPW | Suicidio | Suicide |
| SocPW | Tormento | Torment |
| SocPW | Tradimento | Betrayal |
| SocPW | Tristezza | Sadness |
| PosW | Accord | Agreement |
| PosW | Ambizione | Ambition |
| PosW | Asso | Ace |
| PosW | Astronaut | Astronaut |
| PosW | Attico | Attic |
| PosW | Beatitudine | Bliss |
| PosW | Brezza | Breeze |
| PosW | Commedia | Comedy |
| PosW | Conoscenza | Knowledge |
| PosW | Contante | Cash |
| PosW | Coppia | Couple |
| PosW | Crepuscolo | Twilight |
| PosW | Delizia | Delight |
| PosW | Diamante | Diamond |
| PosW | Diploma | Diploma |
| PosW | Dollar | Dollar |
| PosW | Dono | Gift |
| PosW | Eccitazione | Excitement |
| PosW | Estasi | Ecstasy |
| PosW | Fama | Fame |
| PosW | Fascino | Charm |
| PosW | Firmament | Firmament |
| PosW | Fragranza | Fragrance |
| PosW | Giarrettiera | Garter |
| PosW | Godimento | Pleasure |
| PosW | Idolo | Idol |
| PosW | Impiego | Employment |
| PosW | Incentive | Incentive |
| PosW | Infatuazione | Infatuation |
| PosW | Intelletto | Intellect |
| PosW | Leader | Leader |
| PosW | Liberazione | Liberation |
| PosW | Lusso | Luxury |
| PosW | Melodia | Melody |
| PosW | Milionario | Millionaire |
| PosW | Muffin | Muffin |
| PosW | Nettare | Nectar |
| PosW | Nuotatore | Swimmer |
| PosW | Opinione | Opinion |
| PosW | Orchestra | Orchestra |
| PosW | Orgasm | Orgasm |
| PosW | Ottimismo | Optimism |
| PosW | Passione | Passion |
| PosW | Patriota | Patriot |
| PosW | Possibilità | Possibility |
| PosW | Prateria | Prairie |
| PosW | Prestigio | Prestige |
| PosW | Profitto | Profit |
| PosW | Promozione | Promotion |
| PosW | Ricchezze | Wealth |
| PosW | Ricompensa | Reward |
| PosW | Rinfresco | Refreshment |
| PosW | Rispetto | Respect |
| PosW | Risultato | Result |
| PosW | Salvataggio | Rescue |
| PosW | Scrittore | Writer |
| PosW | Talent | Talent |
| PosW | Trionfo | Triumph |
| PosW | Trofeo | Trophy |
| PosW | Villaggio | Village |
